# QTL mapping pod dehiscence resistance in soybean (*Glycine max* L. Merr.) using specific-locus amplified fragment sequencing

**DOI:** 10.1007/s00122-019-03352-x

**Published:** 2019-06-03

**Authors:** Jianan Han, Dezhi Han, Yong Guo, Hongrui Yan, Zhongyan Wei, Yu Tian, Lijuan Qiu

**Affiliations:** 1 National Key Facility for Gene Resources and Genetic Improvement/Key Laboratory of Crop Germplasm Utilization, Ministry of Agriculture, Institute of Crop Sciences, Chinese Academy of Agricultural Science, No. 12 Zhongguancun South Street, Haidian District, Beijing, 100081 People’s Republic of China; 2 Institute of Soybean Research, Heilongjiang Academy of Agricultural Sciences, Harbin, 150086 People’s Republic of China

## Abstract

**Key message:**

We constructed a high-density genetic linkage map comprising 4,593 SLAF markers using specific-locus amplified fragment sequencing and identified six quantitative trait loci for pod dehiscence resistance in soybean.

**Abstract:**

Pod dehiscence is necessary for propagation in wild soybean (*Glycine soja*). It is a major component causing yield losses in cultivated soybean, however, and thus, cultivated soybean varieties have been artificially selected for resistance to pod dehiscence. Detecting quantitative trait loci (QTLs) related to pod dehiscence is required for molecular marker-assisted selection for breeding new varieties with pod dehiscence resistance. In this study, we constructed a high-density genetic linkage map using 260 recombinant inbred lines derived from the cultivars of Heihe 43 (pod-indehiscent) (ZDD24325) and Heihe 18 (pod-dehiscent) (ZDD23620). The map contained 4953 SLAF markers spanning 1478.86 cM on 20 linkage groups with an average distance between adjacent markers of 0.53 cM. In total, six novel QTLs related to pod dehiscence were mapped using inclusive composite interval mapping, explaining 7.22–24.44% of the phenotypic variance across 3 years, including three stable QTLs (*qPD01*, *qPD05-1* and *qPD08-1*), that had been validated by developing CAPS/dCAPS markers. Based on the SNP/Indel and significant differential expression analyses of two parents, seven genes were selected as candidate genes for future study. The high-density map, three stable QTLs and their molecular markers will be helpful for map-based cloning of pod dehiscence resistance genes and marker-assisted selection of pod dehiscence resistance in soybean breeding.

## Introduction

Pod dehiscence is necessary for spreading seeds of wild plant species that bear seeds in pods and allows wild plants to introduce their progeny into a broad range of environments (Fuller 2007). However, in cultivated crops, seeds from plants that undergo pod dehiscence cannot be harvested, resulting in serious yield losses. During planting and harvesting, resistance to pods dehiscence prior to harvest is critical (Hancock 2004; Harlan et al. 1973). Therefore, during soybean domestication, pod dehiscence resistance was artificially selected as an important trait (Hideyuki et al. 2012). Although researchers intensively screen for and avoid pod dehiscence during crop domestication, pod dehiscence before harvest is still a problem in soybean breeding (Christiansen et al. 2002). Soybean breeding has made great progress in incorporating resistance to pod dehiscence, but little is known about the genetics of pod dehiscence in soybean (Funatsuki et al. 2014). The main limiting factor for genes conferring resistance to pod dehiscence is low marker coverage. This leads to large intervals for detecting and mapping QTLs related to pod dehiscence; a large number of predicted candidate genes are contained within these intervals (Zhang et al. 2016).

The rapid development of soybean genomics research has greatly enhanced the speed and the accuracy of QTL mapping for many important agronomic traits. The completion of the soybean reference genome, Williams 82, has accelerated the construction of a high-density genetic linkage map, allowing for more accurate QTL mapping and gene mining in soybean (Schmutz et al. 2010; Ju et al. 2017; Zhang et al. 2016). In soybean, researchers have constructed many genetic linkage maps using markers: restriction fragment length polymorphisms (RFLPs), simple sequence repeats (SSRs) and single-nucleotide polymorphisms (SNPs) (Gutierrez-Gonzalez et al. 2011; Hyten et al. 2010; Jeong 2002; Keim et al. 1990). To date, multiple studies have identified major QTLs for pod dehiscence in soybean using recombinant inbred lines (RILs) with RFLP (Bailey et al. 1997) and SSR markers (Funatsuki et al. 2006). These studies have shown that pod dehiscence is dominated by several major and minor QTLs. Due to the various genetic backgrounds in different soybean populations, pod dehiscence was found to be controlled by a single recessive major gene or multiple genes (Tsuchiya 1986, 1987; Kang et al. 2005; Funatsuki et al. 2008; Yamada et al. 2009). A major pod dehiscence QTL was discovered on chromosome 16 using RILs, and other minor QTLs were identified on chromosomes 2, 15 and 19 (Bailey et al. 1997). After fine mapping, the candidate gene *Glyma16g25580* was confirmed to control pod dehiscence and was designated as Pod dehiscence 1 (*Pdh1*) (Funatsuki et al. 2014). Meanwhile, a NAC gene named *SHAT1-5*, *Glyma.16g019400*, was found to regulate pod dehiscence in soybean (Dong et al. 2014). Using SSR markers, three novel minor QTLs for pod dehiscence were confirmed on chromosomes 5, 10 and 14 from two sets of RILs (Kang et al. 2009).

Next-generation sequencing (NGS) has provided many sequencing technologies that have been used to construct high-density genetic maps on many populations. NGS techniques include restriction-site-associated DNA tagging sequencing (RAD-seq), genotyping-by-sequencing (GBS) and specific-locus amplified fragment sequencing (SLAF-seq) (Baird et al. 2008; Elshire et al. 2011; Sun et al. 2013). These techniques have become effective tools for QTL mapping and allow researchers to identify, sequence and genotype individuals in a population. Since NGS can directly identify DNA sequence differences with high accuracy, NGS techniques have been widely used for plant and animal genomics (Bhatia et al. 2013). SLAF-seq is a rapid, high-resolution technique based on NGS technology for broad-scale SNP genotyping, which has been extensively used in genomic analyses due to its characteristics of high molecular marker density, large marker number, even distribution, high accuracy and lack of repetitive markers (Sun et al. 2013). This technique can improve the efficiency of QTL mapping associated with important agronomic traits (Cao et al. 2017).

In this study, we selected SLAF markers based on the whole soybean genome through SLAF-seq. Using a set of 260 RILs derived from the cultivars Heihe 43 (ZDD24325) and Heihe 18 (ZDD23620) as parents, a high-density genetic linkage map covering the entire soybean genome was constructed to map QTLs for pod dehiscence. We obtained several QTLs associated with pod dehiscence. The high-density genetic map we generated will facilitate exploration of effective QTLs, and the novel QTLs detected in our population will be helpful for further research on pod dehiscence in soybean.

## Materials and methods

### Plant materials and phenotypic evaluation

We used 260 F_2:7_ recombinant inbred lines (RILs) that were developed by a single-seed descendent (SSD) method from an F_2_ population of the cross between the cultivars Heihe 43 (ZDD24325) and Heihe 18 (ZDD23620) as parents. The two parents and their derived RIL population were grown at the same location of Heihe Experiment Station, Heilongjiang Academy of Agricultural Sciences, from 2015 to 2017. The parents and RIL population were planted four rows per plot (5.0-cm plant spacing, 66.7-cm row spacing and 4.0-m row length). Heihe 43 is resistant to pod dehiscence and is the largest precocious cultivated variety in Heilongjiang province, developed from Heihe 18 and Heihe 23 (ZDD23625) by systematic breeding programs. Heihe 18 is sensitive to pod dehiscence. Although Heihe 18 and Heihe 43 have minimal differences among numerous agronomic traits, the pod dehiscence in Heihe 18 is extremely obvious (Han et al. 2015a).

We used a modified oven-dry method (Kang et al. 2005) to determine the phenotype of pod dehiscence. A total of 30 pods per line per year were carefully collected using scissors at the R8 stage and stored in sealed bags to prevent water evaporation. The pods were then incubated at 80 °C for 5 h in an oven, and the number of dehiscent pods was calculated. The PD was estimated using the following equation: PD = (dehiscent pods number/total pods number) × 100% (Peng et al. 1991).

### Statistical analysis

One-way analysis of variance (ANOVA) was performed by the statistical package SAS version 9.1, including the frequency distribution, the mean of the RIL population, the coefficient of variation (CV), the broad-sense heritability (*h*^2^). The *h*^2^ for PD was estimated using the following equation: *h*^2^ = ${\sigma }_{\mathrm{G}}^{2}$/${\sigma }_{\mathrm{P}}^{2}$ (Nyquist and Baker 1991), where ${\sigma }_{\mathrm{G}}^{2}$ and ${\sigma }_{\mathrm{P}}^{2}$ are the genotypic and the phenotypic variance, respectively.

### DNA extraction

Young, healthy and fresh leaves from both parent lines and all 260 RILs were collected in centrifuge tubes, frozen in liquid nitrogen, ground in a tissue grinder and then stored at − 80 °C. Total genomic DNA was extracted from every leaf sample following the modified CTAB protocol (Saghaimaroof et al. 1984). The quality and concentration of the extracted DNA were assessed by electrophoresis on 1% agarose gels and using a spectrophotometer (UV–Vis Spectrophotometer Q5000).

### SLAF library construction and sequencing

We constructed a SLAF library and sequenced individuals from 260 RILs and their parents. The Williams 82 soybean reference genome sequence (Wm82.a2.v1, https://phytozome.jgi.doe.gov) was used to predict digestion, and we chose a combination of RsaI and HaeIII restriction enzymes to digest the genomic DNA. Amplified fragments ranging from 364 to 414 bp in size were defined as SLAF markers; 132,516 SLAF markers were predicted. A single-nucleotide (A) overhang was added to the obtained digested fragments. Dual-index sequencing adapters were then ligated to the A-tailed fragments. These fragments were generated using PCR and were then purified and mixed. The target fragments were isolated to generate a sequencing library. After purification and dilution, paired-end (each end 125 bp) sequencing was performed on an Illumina HiSeq platform (Illumina, Inc, San Diego, CA, USA). To evaluate the accuracy of the SLAF library, *Oryza sativa* L. *Japonica* was selected as a control for the same treatment and was used in library construction and sequencing. In comparison with the control data, the efficiency of enzyme digestion was evaluated to assess accuracy and effectiveness. Using reads clustering, SLAF markers were developed in parents and their offspring. Polymorphic SLAF markers were screened and used for further genetic map construction.

### SLAF-seq data grouping and genotyping

The procedure of Sun et al. (2013) was adapted for SLAF marker grouping and genotyping of the 260 RILs as follows. The original sequencing read length of the SLAF-seq library was 125 bp. In order to ensure analysis quality, the original sequencing reads were filtered by removing reads containing: (1) barcode adapters; (2) > 10% base content (N); and (3) residue of restriction enzyme fragments. We selected reads ranging from 4 to 103 bp to analyze the data. Filtered reads were aligned to the reference genome using the Burrows–Wheeler Aligner (BWA) (Os et al. 2005), and reads with the same paired end were identified as the same SLAF marker. SLAF markers were analyzed for polymorphism and classification based on the number of alleles and the differences between sequences. SLAF markers were then mapped to the reference genome. The distributions of SLAF markers on chromosomes were used to map SLAF markers and polymorphic SLAF markers on the chromosomes.

Then, all polymorphic SLAF markers for genetic mapping were filtered using the following criteria: (1) average sequence depths of more than tenfold; (2) filtering out SLAF markers with more than five SNPs; and (3) integrity filtering. Markers were required to cover at least 70% of the genotypes in all progeny. Markers with missing data were filtered based on parental genotypes. The final polymorphic SLAF markers were used to construct a high-density genetic map.

### Construction of a high-density genetic map

Polymorphic SLAF markers were aligned with the reference genome and mapped onto 20 chromosomes. We then calculated the MLOD (the modified logarithm of odds) scores between markers and filtered the markers with MLOD values lower than 5. Each chromosome was a linkage group (LG) used as a unit. HighMap software (Li et al. 2008) was used to analyze the linear arrangement of markers in the linkage group and to calculate the map distances. Finally, we obtained a high-density genetic map.

### QTL mapping for pod dehiscence

According to the phenotypes in three environments, QTLs for pod dehiscence were detected using inclusive composite interval mapping (ICIM) in the *R/qtl* package (Broman et al. 2003). A total of 1000 permutation tests at a 95% confidence level were used to set the LOD threshold. Based on 1000 permutations, LOD = 2.5 was used to determine the presence of a putative QTL associated with a target trait in a certain genomic region.

### Gene expression analysis by real-time qPCR (qRT-PCR)

Total RNAs of pods (R6 stage) were extracted from Heihe 43 and Heihe 18, respectively, using TransZol Up Plus RNA Kit (TransGen Biotech). For reverse transcription, the first-strand cDNA synthesis was performed using the TransScript First-Strand cDNA Synthesis SuperMix Kit (TransGen, China). For qRT-PCR, gene expressions were examined using cDNA templates on an Applied Biosystems 7300 Real-Time PCR System. Gene-specific primers for the candidate genes were designed using Primer3.0. The relative gene expression levels followed the ${2}^{-\Delta\Delta\mathrm{Ct}}$ method (Pfaffl 2001). The mRNA level of *GmActin* (*Glyma18g52780*) gene as a reference for normalization and three biological replicates were used for each gene.

### Candidate gene prediction within QTL intervals

Sequences within QTLs were defined according to the Williams 82 soybean reference genome sequence (Wm82.a2.v1, https://phytozome.jgi.doe.gov). The functions of candidate genes were annotated using Blastx program (https://www.geneontology.org/) in Nr (nonredundant), Swiss-Prot and KOG/COG (clusters of orthologous groups). All genes were categorized by Gene Ontology (GO) annotation (https://www.geneontology.org/).

### Development and application of molecular markers within QTLs

According to the different loci within QTLs, we developed CAPS (cleaved amplified polymorphic sequence) and dCAPS (derived cleaved amplified polymorphic sequence) markers and used these molecular markers to detect genotypes of the RILs. The online software dCAPS Finder 2.0 (https://helix.wustl.edu/dcaps/dcaps.html) was used to confirm all enzymes. Within *qPD05-1*, we chose a restriction site and developed a CAPS marker, in which enzyme Mnl I was used as a marker. The *qPD01* QTL has no restriction enzyme sites, so we used the reverse complementary sequence and artificially introduced a mismatch base. With this method, we found a restriction enzyme site to develop a dCAPS marker with HpaII as a candidate marker enzyme. Indel markers were developed based on the Indel sites within *qPD08-1*. PCR primers were designed on both sides of the candidate CAPS/dCAPS markers and the Indel markers using Primer3 software (https://primer3.ut.ee/).

Genomic DNA from parents and 260 RILs were used as templates. The 20-μL PCR contained 100 ng of template DNA, 10 × PCR buffer, 2 mmol/L dNTPs, 2 mmol/L primers and 1 unit of Taq polymerase (Takara). The PCR amplification program was as follows: 95 °C for 4 min, 34 cycles of 95 °C denaturation for 30 s, 58 °C annealing for 40 s, 72 °C extension for 1 min and a final 72 °C extension for 10 min. PCR products were detected using 0.8% agarose gel electrophoresis. In accordance with the restriction enzyme digestion method (New England BioLabs, NEB), the 10-μL system consisted of 5 μL of PCR product, 0.2 μL 10 U/μL of enzyme, 1.5 μL of buffer (NEB, www.neb.com/) and 3.3 μL of ddH_2_O and was digested at 37 °C in a water bath for 40 min. The digested products of CAPS/dCAPS were detected using 7% polyacrylamide gel electrophoresis, and PCR products of Indels were denatured and then separated using 7% denaturing polyacrylamide gel electrophoresis (PAGE).

## Results

### Phenotypic analysis of pod dehiscence

**Fig. 1:**
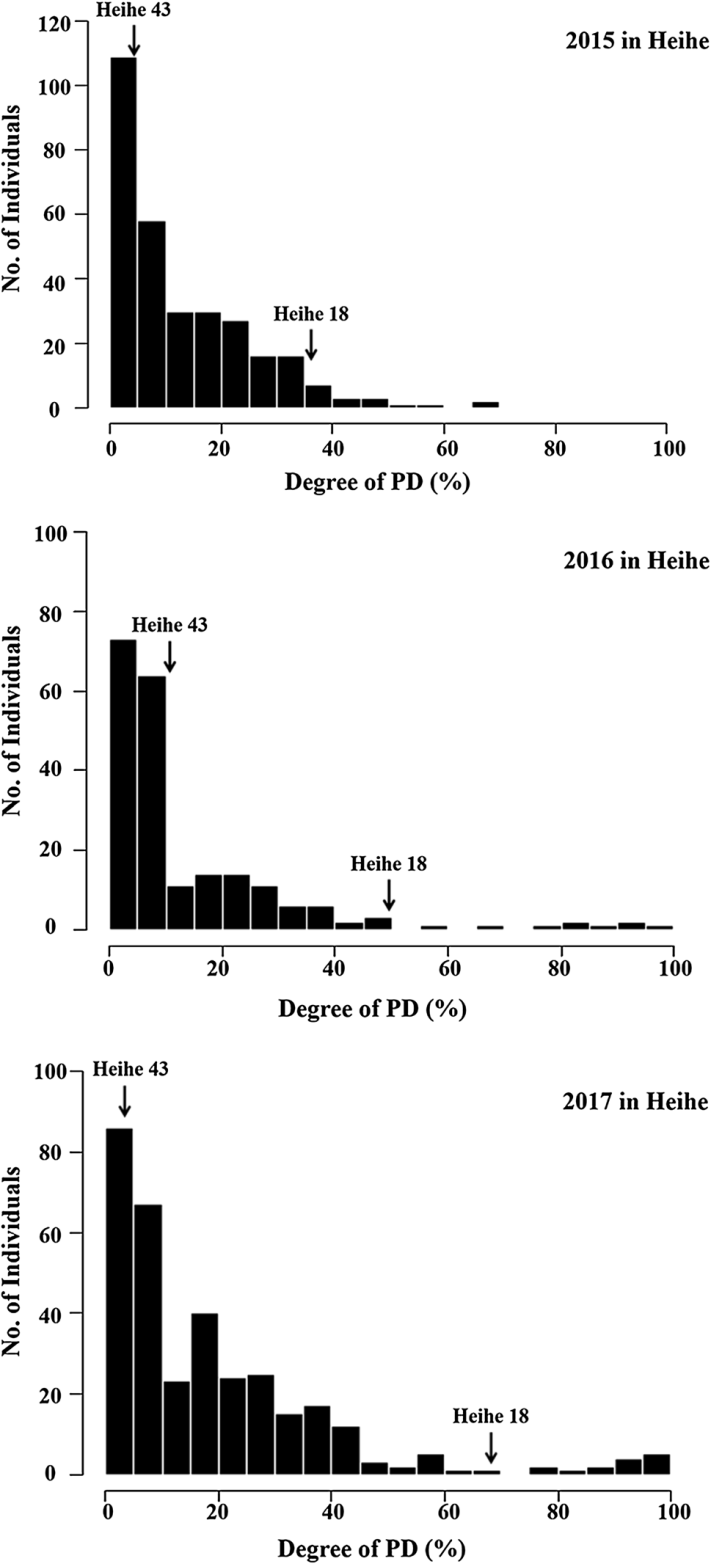
Frequency distributions of pod dehiscence in RILs from 2015 to 2017. The cultivars Heihe 43 and Heihe 18 indicated by the arrows designate the degree of pod dehiscence in parental lines

We identified the PD of parents and the RILs in Heihe, Heilongjiang province, in 2015, 2016 and 2017 (Fig. 1 and Table 1). Heihe 18 had higher PD than Heihe 43 at 37% and 3% in 2015, 47.03% and 11.36% in 2016 and 75% and 3.33% in 2017. RIL population showed more resistance of pod dehiscence over 3 years, showing that the female Heihe 43 contributed to resistance of pod dehiscence and the positive skewness indicated a transgressive segregation toward lower PD. The broad-sense heritability of pod dehiscence was 81.3% in our population.

**Table 1:** Statistical analysis of pod dehiscence in the parents and the RIL population in 3 years in Heihe

Year	Parents		RILs					
	Heihe 43	Heihe 18	Mean	SD	Range	Skewness	Kurtosis	CV (%)
2015	3	37	9.9	11.7	0–68.8	2	5.6	1.2
2016	11.4	47	11.2	18.9	0–100	3.6	13	1.7
2017	3.3	75	16.2	22	0–100	2.4	5.7	1.4

### SLAF-seq and genotyping of RILs

**Fig. 2:**
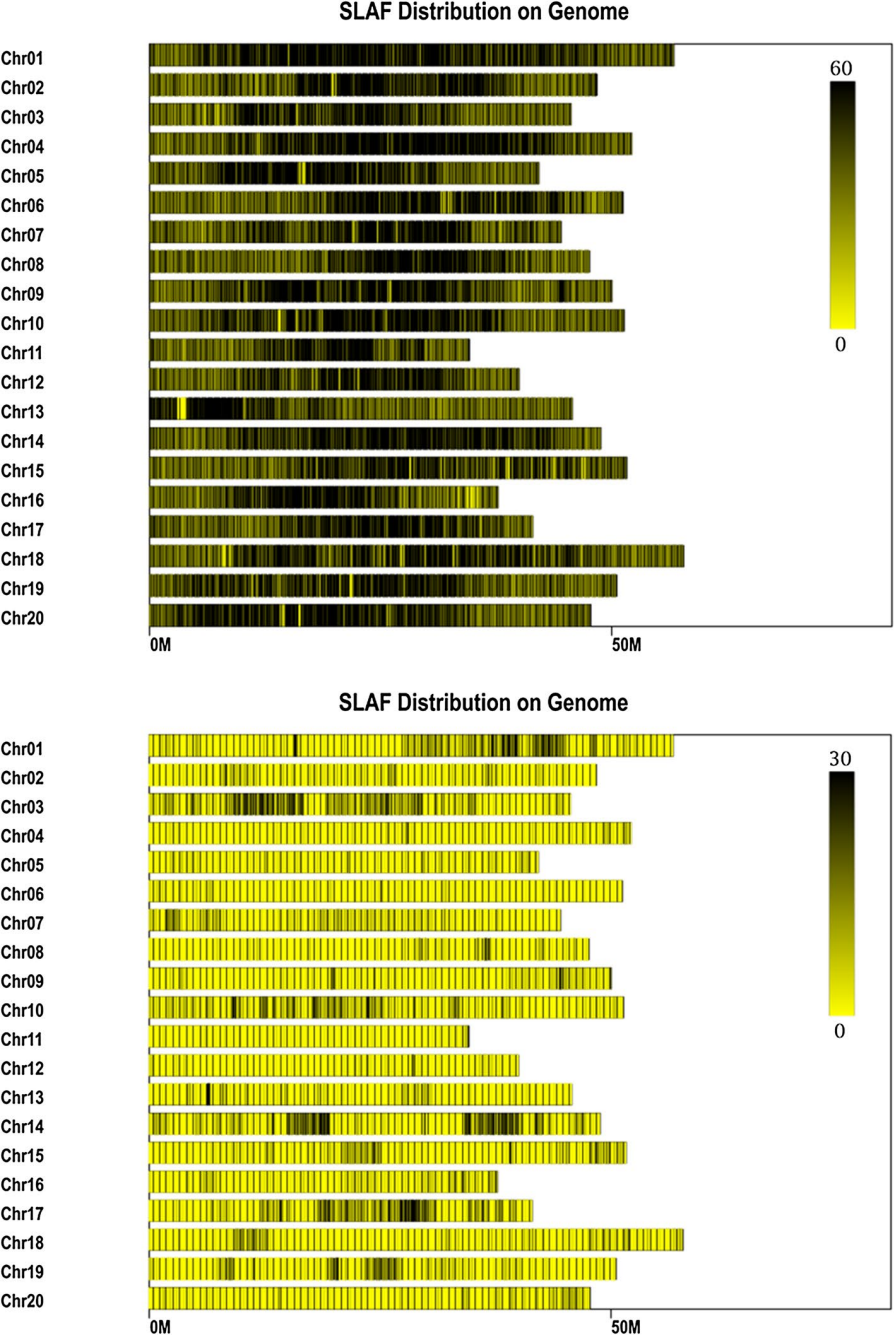
Distribution of SLAF markers and polymorphic SLAF markers on chromosomes. The abscissa is the length of the chromosome, and each yellow stripe represents a chromosome. The genomes were divided in units of 1 M. Different colors indicate the number of SLAFs, the color is deeper and the SLAFs are more. Darker regions in the figure are concentrated distributions of SLAF markers. The upper panel of the figure represents the distribution of the SLAF tag and the bottom panel of the figure represents the distribution of polymorphism of SLAF markers (with M as the unit)

The parent lines and RILs were sequenced and genotyped using SLAF-seq. The sequence depths in Heihe 43 and Heihe 18 were 39.74 × and 34.87 × , respectively, and the number of SLAF markers was 195,279 and 205,644, respectively. The average sequence depth was 12.35 × . The enzyme digestion efficiency was 89.82%. There were 398,386 controlled sequencing reads that were used to evaluate the accuracy of library construction; these reads were compared with the reference soybean genome. The alignment efficiency was 86.29%. The average Q30 of sequencing was 80.40%, and the average GC content was 38.12%. In total, we obtained 364,461 SLAF markers, of which 24,249 were polymorphic and the proportion of polymorphisms was 6.65%. After filtering and quality assessment, 4593 SLAF markers were used to construct a high-density genetic map. Chromosomal distribution of all SLAF markers and polymorphic SLAF markers was plotted according to the distribution of SLAFs on the chromosomes (Fig. 2). Compared with the soybean reference genome, we identified a total of 6834 SNPs, of which 4576 were transition type (Tri) and 2258 were the transversion type (Trv) (Table 2).

**Table 2:** The SNP information of the mapped SLAF markers

Chr^a^	SNP number^b^	Trv^c^	Tri^d^	Trv/Tri (%)
1	759	226	533	0.42
2	93	43	50	0.86
3	1049	347	702	0.49
4	125	49	76	0.64
5	163	52	111	0.47
6	87	33	54	0.61
7	512	180	332	0.54
8	187	70	117	0.6
9	150	52	98	0.53
10	682	212	470	0.45
11	50	15	35	0.43
12	22	6	16	0.38
13	170	63	107	0.59
14	723	241	482	0.5
15	566	176	390	0.45
16	390	146	244	0.6
17	568	174	394	0.44
18	279	80	199	0.4
19	131	47	84	0.56
20	128	46	82	0.56
Total	6834	2258	4576	0.49

^a^Chr: Chromosome

^b^Trv: Transversion-type SNP

^c^Tri: Transition-type SNP

^d^Trv/Tri: Transversion-type SNP/transition-type SNP

### Construction of a high-density genetic map in soybean

**Fig. 3:**
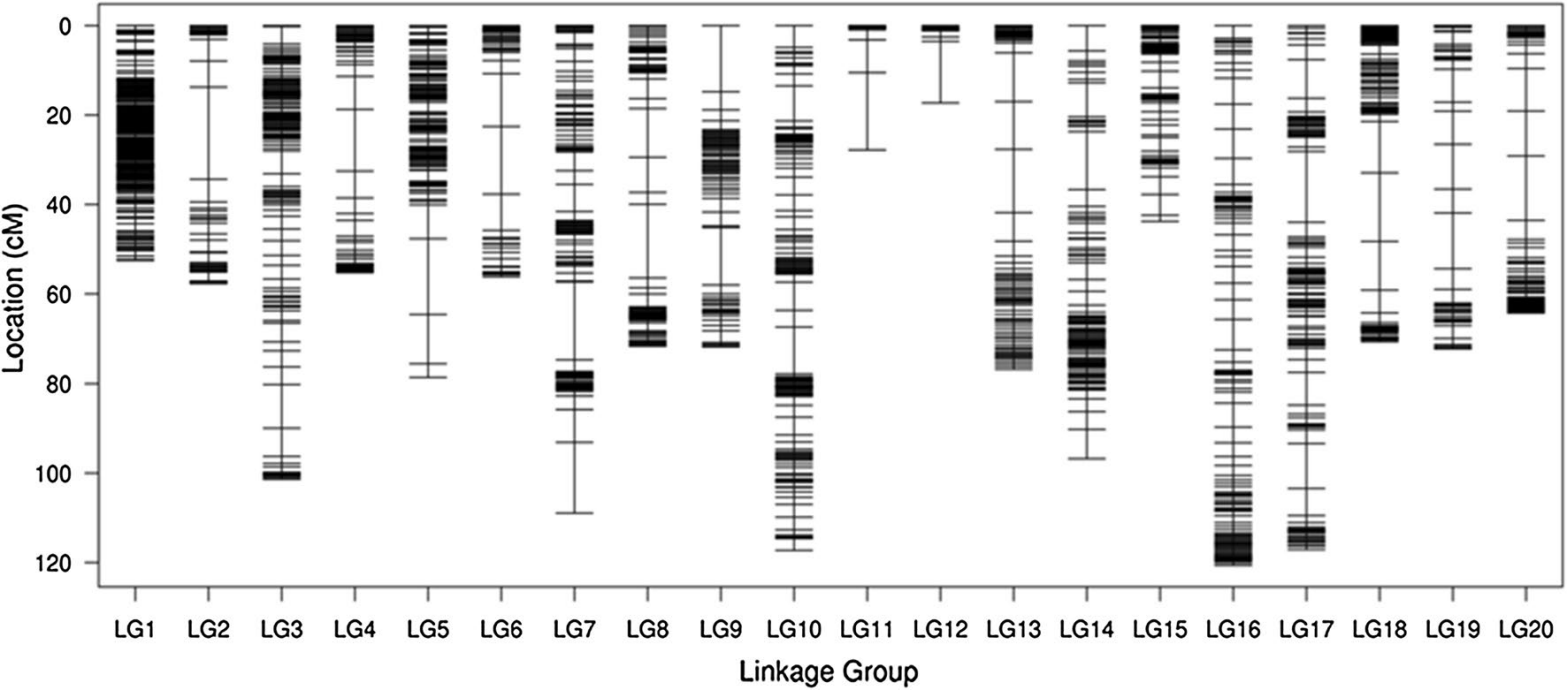
High-density linkage map. SLAF markers are distributed on 20 chromosomes. The black bars in each linkage group represent mapped SLAF-seq markers. The linkage group number is shown on the *x* axis, and genetic distance is shown on the *y* axis (cM is the unit)

**Fig. 4:**
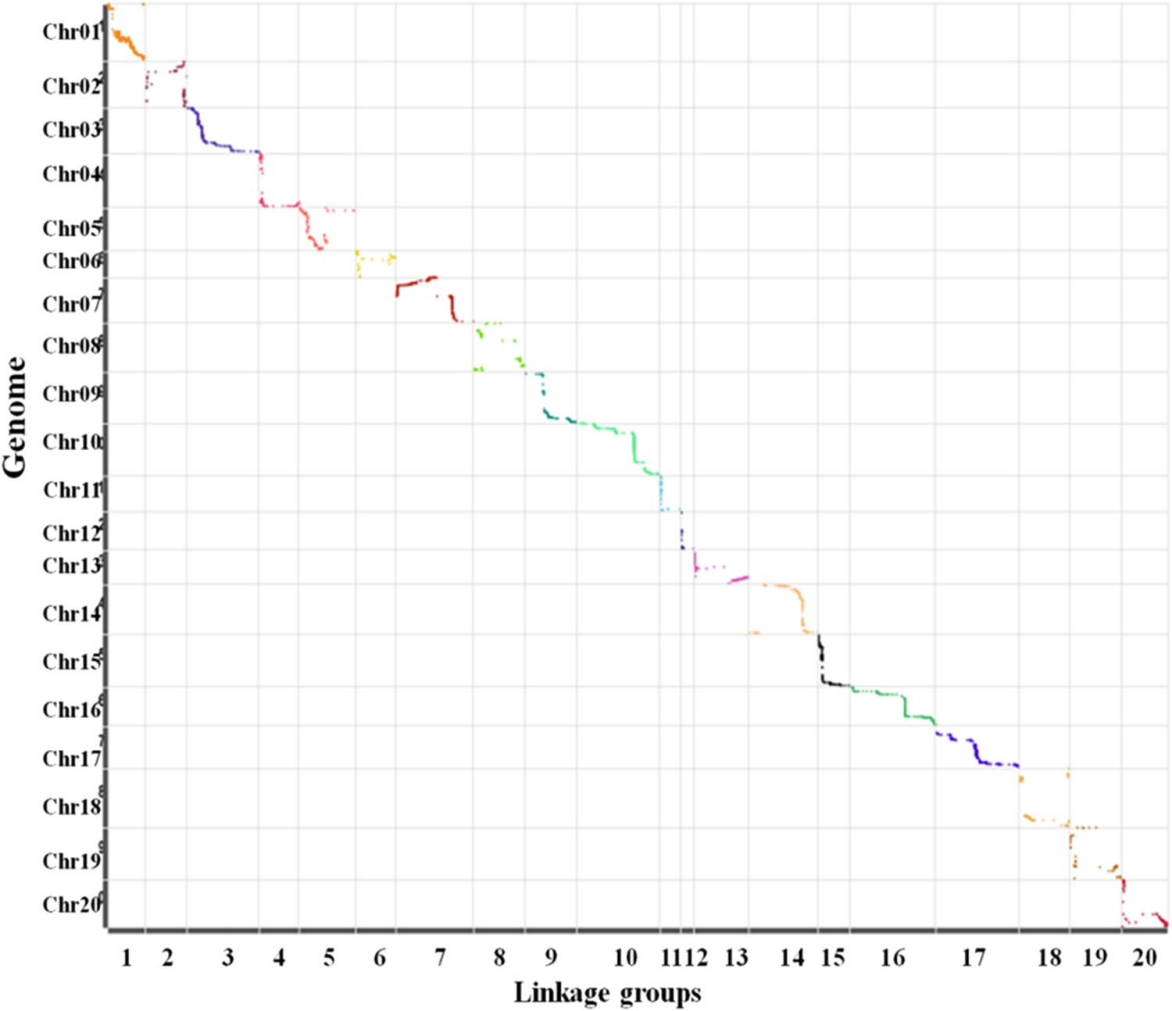
Collinearity analyses of the genetic map and genome. The abscissa is the genetic distance of each linkage group; the ordinate is the physical length of each linkage group, which scatters the form of markers in the genome and genetic map collinearity. Different colors represent different chromosomes or linkage groups

A total of 4593 polymorphic markers were mapped on 20 chromosomes based on the reference genome, and a high-density genetic map was constructed, with a total length of 1478.86 cM and an average distance between markers of 0.53 cM (Fig. 3). The mean chromosome length was 73.94 cM. Seven hundred and six markers were assigned on chromosome 3 with a genetic length of 101.35 cM, which was the longest of all chromosomes. Seventeen markers were assigned on chromosome 12 with a genetic length of 17.27 cM, which was the shortest of all chromosomes. The minimum number of markers was consistent with its physical length (Schmutz et al. 2010). The proportion of gaps < 5 cM between two markers was 95.72% (Table 3). Collinearity analysis (Fig. 4) of the position of markers and the genetic map on the genome showed that the order of markers on the 20 chromosomes was consistent with the genome, indicating high collinearity, indicating that the gene annotation within QTL intervals was reliable.

**Table 3:** Characteristics of the high-density genetic map

Chr^a^	No.Markers^b^	Genetic distance (cM)	Avg. distance between markers (cM)	Gaps ≤ 5 (%)	Max.gap (cM)
1	489	52.51	0.11	100	2
2	66	57.71	0.89	95.38	20.61
3	706	101.35	0.14	99.57	9.75
4	103	55.26	0.54	96.08	13.8
5	135	78.59	0.59	97.76	16.95
6	64	56.2	0.89	95.24	15.12
7	393	108.94	0.28	97.19	17.33
8	115	71.71	0.63	97.37	16.47
9	96	71.87	0.76	97.89	14.79
10	463	117.27	0.25	99.35	10.38
11	44	27.83	0.65	93.02	17.33
12	17	17.27	1.08	87.50	13.75
13	111	76.76	0.7	82.73	14.16
14	450	96.77	0.22	99.11	12.97
15	338	43.79	0.13	100	4.63
16	272	120.6	0.45	97.79	6.81
17	399	117.1	0.29	98.74	15.84
18	157	70.73	0.45	97.44	15.34
19	84	72.31	0.87	85.54	12.45
20	91	64.29	0.71	96.67	14.41
Total	4593	1478.86	0.53	95.72	20.61

^a^Chr. indicates chromosome

^b^No. markers, the number of markers on chromosome

### QTL for pod dehiscence

**Fig. 5:**
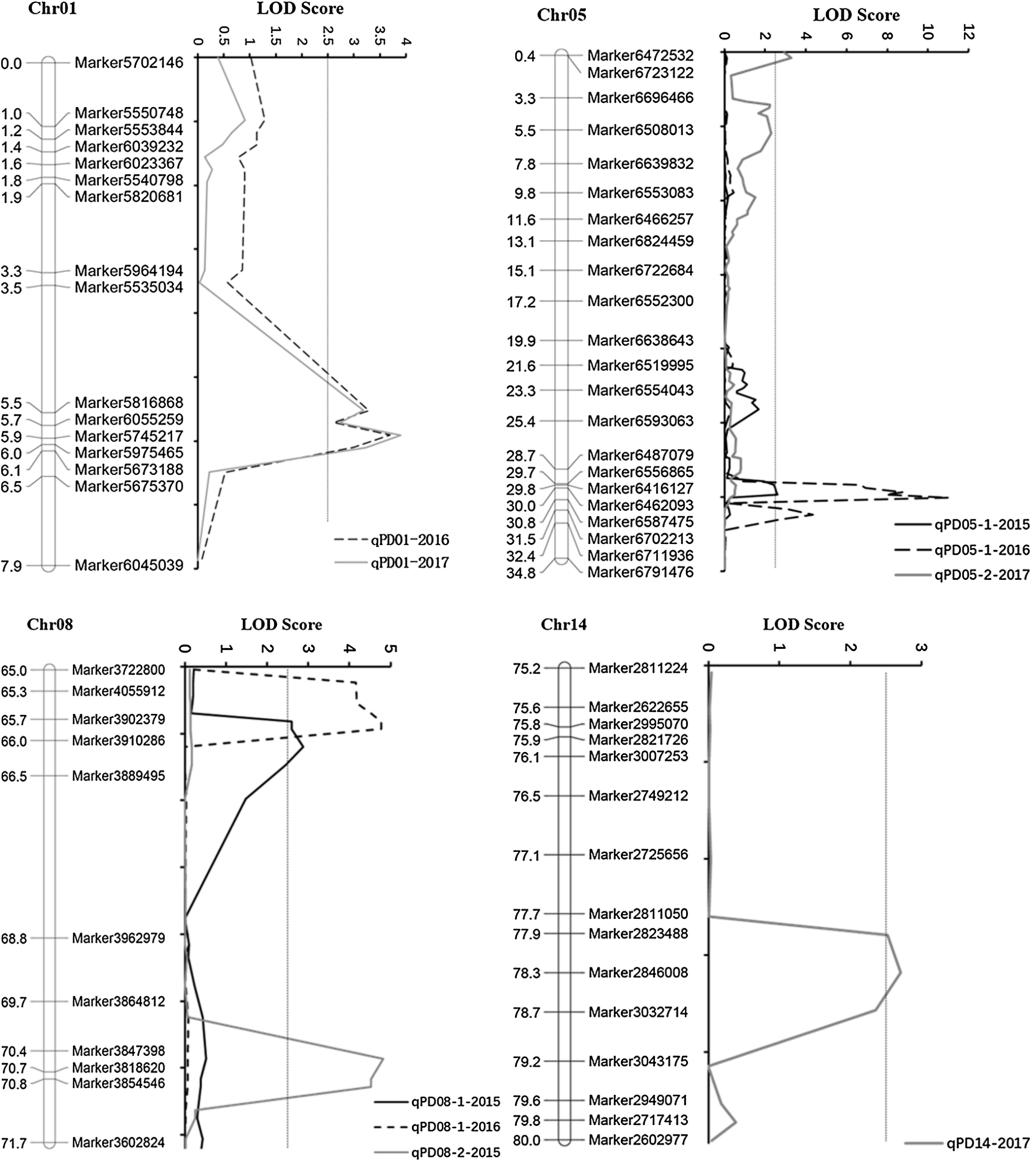
Mapping of QTLs for pod dehiscence on Chr01, Chr05, Chr08 and Chr14. The curves indicate the physical position of markers against LOD score of QTL detected on chromosomes. Different lines represent different years

The *R/qtl* package was used to identify QTL associated with pod dehiscence in 3 years. In total, six novel QTLs were detected and were located on chromosomes 1, 5, 8 and 14 (Fig. 5). All of QTLs were not found in previous studies; thus, these QTLs could be considered as novel QTLs for pod dehiscence. The LOD score of these QTLs ranged from 2.61 to 11.00, and they could explain 7.22% to 24.44% of the phenotypic variation (Table 4). Of these, four QTLs (*qPD01*, *qPD05-1*, *qPD05-2* and *qPD08-1*) explained high phenotypic variations ( > 10%). In addition, *qPD01*, *qPD05-1* and *qPD08-1*, distributed on chromosomes 1, 5 and 8, respectively, could be detected in different years. Thus, these three QTLs might be the major and stable QTLs in our population. The additive effect of these QTLs was negative, which showed that the positive alleles came from the female Heihe 43 and the positive alleles improved the resistance of pod dehiscence.

**Table 4:** QTL for pod dehiscence in soybean across three environments

Env^a^	Name^b^	Chr^c^	Flanking marker^d^	Genetic interval^e^	Physical_interval	^f^LOD	^g^PVE	^h^ADD
2015	*qPD05-1*	5	mk6556865-mk6416127	29.65–29.85	40,655,498–40,703,417	2.61	11.19	− 6.00
	*qPD08-1*	8	mk4055912-mk3902379	65.32–65.70	35,608,667–38,482,164	2.88	10.86	− 3.53
	*qPD08-2*	8	mk3847398-mk3854546	70.36–70.79	42,569,027–42,571,686	4.82	8.37	− 0.44
2016	*qPD01*	1	mk5816868-mk5975465	5.52–5.91	5,149,656–5,408,961	3.94	24.44	− 14.21
	*qPD05-1*	5	mk6556865-mk6462093	29.65–30.04	40,448,596–40,703,417	11.00	15.14	− 0.52
	*qPD08-1*	8	mk3902379-mk3910286	64.74–65.70	35,560,353–38,482,164	6.18	19.61	− 12.72
2017	*qPD01*	1	mk5816868-mk5673188	5.52–6.10	5,149,255–14,522,888	3.90	21.37	− 15.12
	*qPD05-2*	5	mk6723122-mk6472532	0.39	484,893–496,307	3.29	17.57	− 13.71
	*qPD14*	14	mk2823488-mk2846008	77.89–78.28	45,997,368–46,185,304	2.71	7.22	− 0.46

^a^Env: the three specific environments are designed as follows: 2015, 2016, 2017

^b^The name of each QTL is a composite of pod dehiscence

^c^Chr: chromosome

^d^Flanking markers: the markers to the left and right of the QTL

^e^Interval: the interval of confidence in centimorgan

^f^LOD: the logarithm of odds score

^g^PVE: the phenotypic variance explained by individual QTL

^h^ADD: the additive effect value

### Candidate genes prediction and expression analysis

**Fig. 6:**
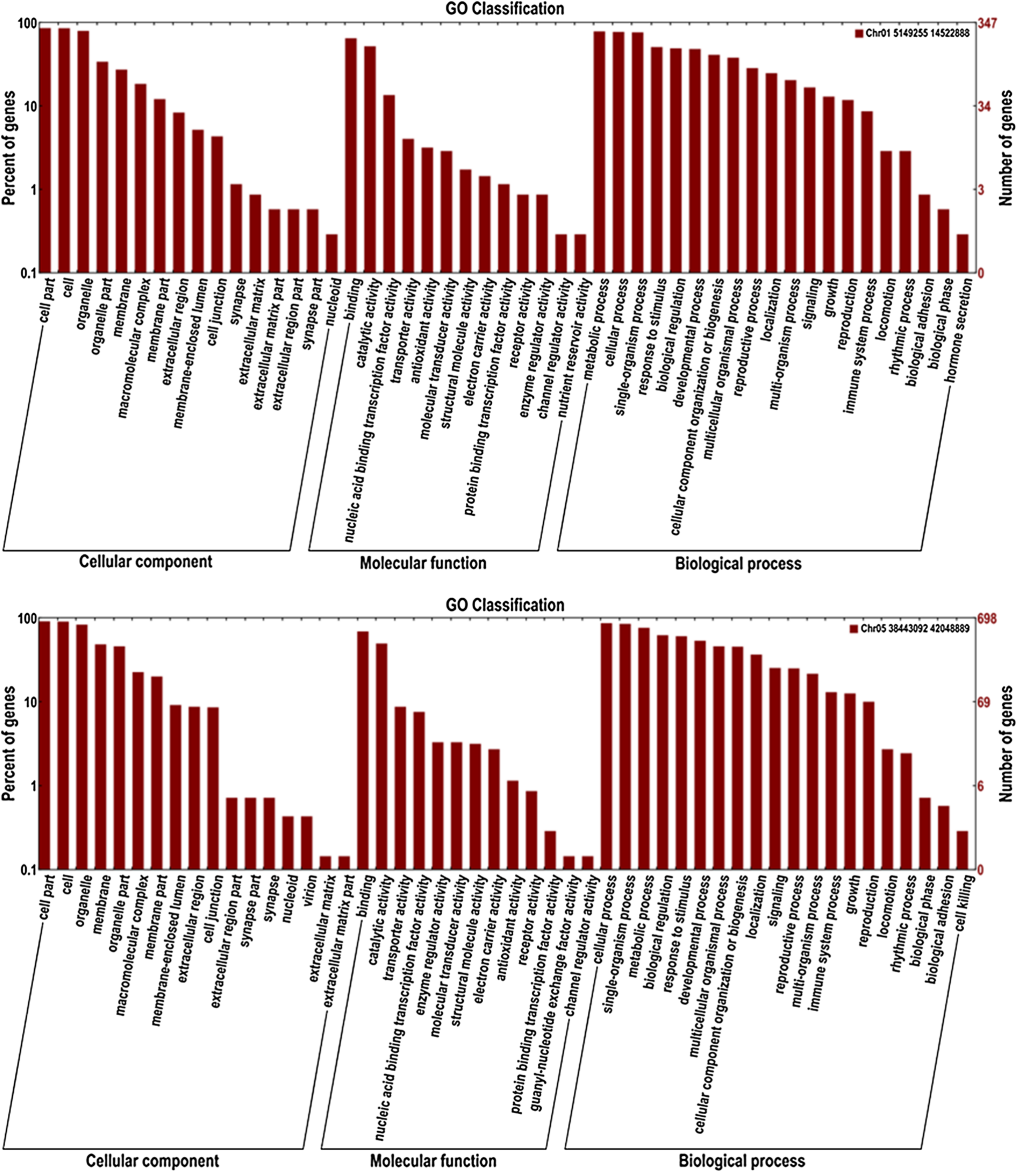

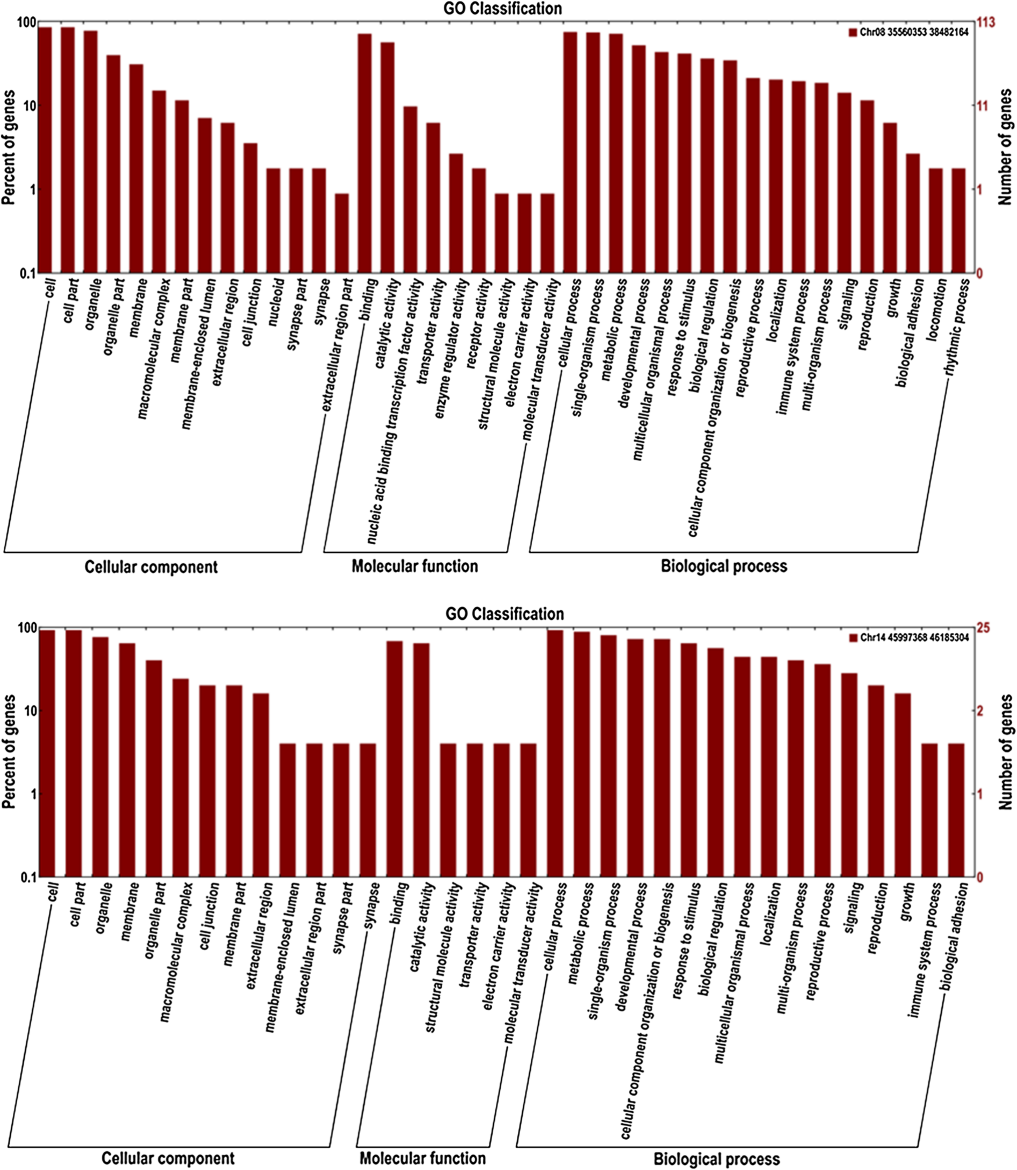
Gene ontology (GO) annotation of genes within the QTLs. The *y* axis on the right shows the number of genes in each category, and the *y* axis on the left shows the percentage of a specific category of genes in that main category

**Fig. 7:**
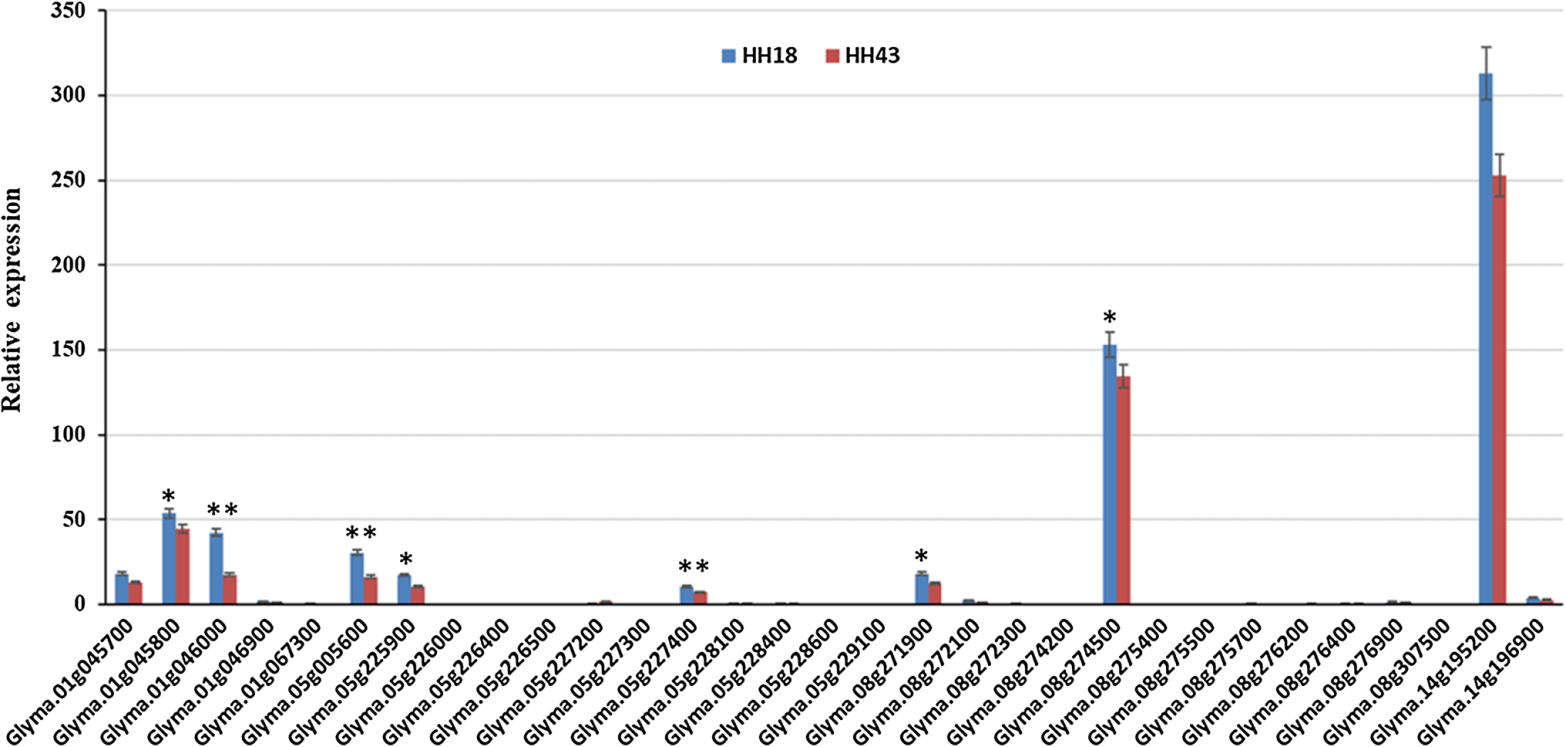
Expression level of 31 candidate genes. *Y* axes indicate the ratios of relative fold expression levels. Asterisks indicate significant differences as determined by ANOVA (**P < 0.01). Relative expression was calculated based on the expression level of the *GmActin* gene

Based on the Williams 82 soybean reference genome, six QTL intervals contained 639 genes located on chromosomes 1, 5, 8 and 14. Currently, only 217 genes functions have been annotated by GO annotation (Fig. 6). To further select candidate genes, we compared all these sequence variations within the QTL intervals and screened out 191 SNPs and 23 Indels in 34 genes that were different between the two parents (Table 5). Of 34 genes, 32 genes were annotated their functions (Table 6). Except three genes without suitable primers to be tested, using gene-specific primers for the candidate genes (Table 7), the expression levels of the 31 candidate genes (Fig. 7) were investigated in the pods (R6 stage) collected from Heihe 43 and Heihe 18 by qRT-PCR and different levels of transcription abundance were observed among these genes. Among 31 genes, nine genes had relatively high expression levels, including *Glyma.01g045700*, *Glyma.01g045800*, *Glyma.01g046000*, *Glyma.05g005600*, *Glyma.05g225900*, *Glyma.05g227400*, *Glyma.08g271900*, *Glyma.08g274500* and *Glyma.14g195200*. Seven of nine genes (*Glyma.01g045800*, *Glyma.01g046000*, *Glyma.05g005600*, *Glyma.05g225900*, *Glyma.05g227400*, *Glyma.08g271900* and *Glyma.08g274500*) had significant differences between two parents.

**Table 5:** SNP and Indel information of the QTL interval

Location	SNP number				Indel number			
	Chr1	Chr5	Chr8	Chr14	Chr1	Chr5	Chr8	Chr14
Intergenic	24	1	104	0	4	0	8	0
Upstream	7	6	3	0	2	1	1	0
5′UTR	1	0	0	0	0	0	0	0
Intron	0	1	7	0	0	0	1	0
Codon insertion	0	0	0	0	0	1	0	0
Nonsynonymous	1	1	2	0	0	0	0	0
Synonymous	1	1	0	0	0	0	0	0
3′UTR	0	0	0	0	0	1	0	1
Downstream	6	7	11	7	0	0	3	0
Total	40	17	127	7	6	3	13	1

**Table 6:** Information of candidate genes

Gene ID	Position	Reference^a^	Alt^b^	Heihe 18	Heihe 43	Effect	Functional annotation
*Glyma.01G045700*	5,261,304	G	A	A	G	Downstream	Ran guanine nucleotide release factor
	5,261,546	A	T	T	A	Downstream	
	5,261,564	A	G	G	A	Downstream	
	5,265,412	C	A	A/C	C	Downstream	
	5,265,632	C	T	C/T	C	Downstream	
*Glyma.01G045800*	5,281,093	C	T	T	C	Nonsynonymous	Peroxisomal adenine nucleotide transporter
	5,281,103	G	A	A	G	Synonymous	
*Glyma.01G046000*	5,317,113	T	37 bp^c^	37 bp^c^	—	Upstream	Fatty acid/sphingolipid desaturase
	5,317,124	T	C	C	—	Upstream	
	5,317,340	T	A	A	—	5′UTR	
*Glyma.01G046900*	5,472,780	T	C	C	T	Upstream	Disease resistance protein (TIR–NBS–LRR class) family
	5,473,035	C	T	T	C	Upstream	
	5,473,041	G	T	T	G	Upstream	
	5,473,047	A	G	G	A	Upstream	
*Glyma.01G066400*	10,415,142	G	A	A	G	Downstream	Subtilisin-like serine endopeptidase family protein
*Glyma.01G067300*	10,968,320	G	A	G	A	Upstream	RNA polymerase II large subunit
	10,968,399	ATGTC	A	ATGTC	A	Upstream	
	10,968,692	C	T	C	T	Upstream	
*Glyma.05G005600*	493,505	C	A	A	C	Synonymous	DNA (cytosine-5)-methyltransferase
*Glyma.05G225900*	40,448,939	G	A	G	A	Upstream	Encodes oxidative stress 3
*Glyma.05G226000*	40,458,086	ACTCT	A	ACTCT, A	A, A	Upstream	Pectin lyase-like superfamily protein
*Glyma.05G226300*	40,473,930	T	C	C/T	C	Upstream	Encodes a chloroplast-localized sulfate transporter
*Glyma.05G226400*	40,485,078	A	G	—	G	Upstream	MATE efflux family protein
*Glyma.05G226500*	40,492,397	C	T	C/T	T	Upstream	—
*Glyma.05G227200*	40,542,215	A	ACTG	ACTG, ACTG	—	Codon insertion	Agamous-like 29
	40,542,235	T	G	G	—	Nonsynonymous	
*Glyma.05G227300*	40,556,233	T	A	A	—	Upstream	Type I MADS domain protein
*Glyma.05G227400*	40,556,283	G	A	A	—	Downstream	NADP-dependent malic enzyme 1
	40,556,523	T	C	C	—	Downstream	
	40,556,559	T	A	A	—	Downstream	
*Glyma.05G228100*	40,620,539	C	T	T	—	Intron	Ethylene-responsive transcription factor
*Glyma.05G228400*	40,638,320	C	T	T	—	Downstream	Sugar transport protein 14
*Glyma.05G228600*	40,655,786	G	A	A/G	G	Downstream	hypothetical protein MTR
	40,655,818	G	A	A/G	G	Downstream	
	40,655,826	A	G	A/G	A	Downstream	
*Glyma.05G229100*	40,696,165	A	G	G	A	Upstream	TCP interactor-containing ear motif protein 1
*Glyma.08G271900*	35,602,669	T	C	C	T	Upstream	MYC-related transcriptional activator with a DNA-binding domain
	35,602,690	T	C	C	T	Upstream	
*Glyma.08G272100*	35,634,072	G	A	A	—	Intron	VQ motif
*Glyma.08G272300*	35,681,077	C	T	T	C	Upstream	Polyamine uptake transporter 4
*Glyma.08G274200*	36,207,423	A	ATGAC	A, ATGAC	A, A	Downstream	Helix-loop-helix DNA-binding domain (HLH)
*Glyma.08G274500*	36,346,605	T	C	C	T	Downstream	Oxidoreductase activity
*Glyma.08G275300*	36,438,490	G	T	T	G	Downstream	PIF1 helicase
*Glyma.08G275400*	36,545,910	A	T	T	—	Upstream	Concanavalin A-like lectin protein kinase family protein
*Glyma.08G275500*	36,601,064	C	T	T	C	Upstream	Rhamnogalacturonate lyase family protein
	36,607,697	C	T	T	C	Intron	
*Glyma.08G275700*	36,661,612	C	A	A	C	Nonsynonymous	Elicitor-activated gene 3–2
	36,661,915	A	AG	AG, AG	A, A	Intron	
	36,661,938	A	G	G	A	Intron	
	36,661,960	G	C	C	G	Intron	
	36,667,791	G	A	A	G	Downstream	
	36,668,120	T	C	C	T	Downstream	
	36,668,144	T	C	C	T	Downstream	
	36,668,167	T	C	C	T	Downstream	
*Glyma.08G276200*	36,839,140	C	T	T	—	Downstream	—
	36,839,156	T	C	C	—	Downstream	
*Glyma.08G276400*	36,941,543	G	A	A	—	Intron	Aldehyde dehydrogenase 6B2
	36,941,840	A	G	G	—	Intron	
	36,962,396	T	C	C/T	T	Nonsynonymous	
*Glyma.08G276900*	37,018,335	A	G	G	—	Intron	tRNA/rRNA methyltransferase (SpoU) family protein
*Glyma.08G307500*	42,571,657	C	G	G	C	Downstream	SAUR-like auxin-responsive protein family
*Glyma.14G195200*	46,052,403	T	G	G	T	Downstream	MAPK phosphatase that negatively regulates MPK4 and MPK6
	46,052,441	T	G	G	T	Downstream	
	46,052,453	C	T	T	C	Downstream	
	46,052,461	G	A	A	G	Downstream	
	46,052,732	T	C	C	T	Downstream	
*Glyma.14G196900*	46,184,758	T	TA	TA	T	3′UTR	Glutaredoxin family protein
	46,185,029	C	A	C	A	Downstream	
	46,185,219	T	C	T	C	Downstream	

^a^Reference indicates the genotype of Williams 82

^b^Alt indicates the genotype of alter

^c^37 bp indicates 37 bp insert, TTAAAACATTTTATAATTTTTTCTACATTTTTTTCCA

**Table 7:** Primer sequences for qRT-PCR

Gene ID	Sequence of primer (5′–3′)	Size (bp)
*Glyma.01g045700*	F: TGATGTTGCTGATAATGGGAGT	246
	R: TCAACTCCTCTAAGACGCAAAT	
*Glyma.01g045800*	F: GAACGTGGATCTGGAATCATTG	136
	R: TTTTATTCGACCTGAGGAACGA	
*Glyma.01g046000*	F: TTTCAACAAGGTTGCACAGATC	232
	R: GCAGATCAAGAACCTAGCAATG	
*Glyma.01g046900*	F: AATGCTTAAAACATCAGGGCAG	87
	R: CATAACTTCCTTTCTGATGGCG	
*Glyma.01g067300*	F: TGTGATATCGGGGAGCTGGTTGA	129
	R: CTGTTTCCTTTTGATGGGTTCAGGA	
*Glyma.05g005600*	F: GGATGATGTAAGGGAGCTAGAC	214
	R: TCATCGATATTTGGCCGACATA	
*Glyma.05g225900*	F: GCAACTTTTGTATCCGTGCTAA	182
	R: GACTTCTTCGTGTGAGAAAAGC	
*Glyma.05g226000*	F: TGACACAAGCCAATTTACACAG	253
	R: CACCCATTTCATGAACTGTTGT	
*Glyma.05g226400*	F: GAACATCACAGGTTATTCGGTG	167
	R: TGTTGAGCCAAAGGAGTGATAT	
*Glyma.05g226500*	F: AAGACTTGATGCTGGGCTGGTGG	95
	R: GTTTTATTTTGTTGTCCCTGTGG	
*Glyma.05g227200*	F: AGAGTGAAGGACTATGTCAACG	202
	R: TTCAGTGTCATCTCACTCTACG	
*Glyma.05g227300*	F: GTCCAAGGAACATCTAATGAAGCTG	132
	R: CACTCCTTTTTGTTTCATTCTCTCG	
*Glyma.05g227400*	F: GGCTCTAACAAGGATGTGTTTC	182
	R: GCAAATGAAACGACTTGAGTCA	
*Glyma.05g228100*	F: GCGTCACTGTCGCAGTCGTCATC	147
	R: CTCCCCACTCGGGTCGGGTATTT	
*Glyma.05g228400*	F: TGCTTGTTGGAGGTATTTTGTG	129
	R: GATCCTCAAATTCAGCTTCGAC	
*Glyma.05g228600*	F: AATAATTCTAATACCTTTTTACC	95
	R: CAAGAGTCTGATTCTGACATCTC	
*Glyma.05g229100*	F: TACTCATCATCATACGCAGCTT	120
	R: CCATCTTGCTGTATTAGTTGGC	
*Glyma.08g271900*	F: CATTTTTCAGAACCCCGATCTG	229
	R: GGTTTCGAAATGCATCGTCTTA	
*Glyma.08g272100*	F: TTCTCCAACCTCTCAGTTTCTC	84
	R: AACAGTGGTCGATAACAAGACT	
*Glyma.08g272300*	F: TATCAGACTCAATGGGAAAACGG	103
	R: TTGCAGATAAAACCGCGCCAAAA	
*Glyma.08g274200*	F: GTATCAGCAGCATACCCTTTTG	203
	R: CTGTCTTTCTCCTCAACTTTGC	
*Glyma.08g274500*	F: GCAGTTGTTGCAATTGGAAGATGTT	93
	R: GCACCACTCACAGGTAGGGATAG	
*Glyma.08g275400*	F: GAATCCGCATTCAACATGAAGA	117
	R: GGATTGAATGGAGTTGACATCG	
*Glyma.08g275500*	F: AAAGGTTTTTGGCCCTGTATTC	128
	R: TAGGAAAATCGTAAGGCCAACT	
*Glyma.08g275700*	F: TAGGAGACAAGGTGGGTGTGGGA	167
	R: GCTATACACTGTGATCCCAGCACAG	
*Glyma.08g276200*	F: TCGTGATGTGGTTATTTGAACAA	113
	R: AAGGCGAAGAGATGGAAGAGAGT	
*Glyma.08g276400*	F: TGATGTTATAAATCCCGCAACG	84
	R: GCACTAACTGCAGCTTTAAACT	
*Glyma.08g276900*	F: GTGCTTTAAGCCAAGTCTTTCA	90
	R: GTTATCGGCTTCGGTTTTTCTT	
*Glyma.08g307500*	F: AATAGTGCTCTCTCTCACACAC	193
	R: GTGCTCAAACTTGCAGTTGTAA	
*Glyma.14g195200*	F: TATTGCAGTTGAGATCGCATTG	167
	R: TAATATCCCCCTCTCCCCTATG	
*Glyma.14g196900*	F: CTTAAGAAGATCCTCCTGGACC	187
	R: GTAAACAAGAGGGGCACTTAAC	
*GmActin*	F: GGTGGTTCTATCTTGGCATC	246
	R: CTTTCGCTTCAATAACCCTA

### Development of molecular markers

According to different sites within the QTL interval, PCR amplifications were conducted using the designed primers (Table 8), and we obtained single PCR products with the same size as the target fragments. Based on alignment with Williams 82 reference sequence, there are differences between the two parents, which was consistent with the prediction.

**Table 8:** Information of molecular markers developed within QTLs

QTL	Marker type	Allele 1/Allele 2	Position	Enzyme	Sequence of primer (5′–3′)	PCR product (bp)	After digestion-bp
							Allele 1/Allele 2
*qPD01*	dCAPs	T/C	5,281,093	Mnl I	F: GATTCCAGATCCACGTTCATCTCCT	157	157(T)/132 + 25(C)
				(CCTC)	R: TCCTTCCCTTGTCTCATTACTCC		
*qPD05-1*	CAPs	A/G	40,448,939	HpaII	F: CCTAGCTATTTCATCTTCACGA	540	540(A)/294 + 246(G)
				(CCGG)	R: AATCCTTACAACGTACGTGTGT		
*qPD08-1*	Indel	GTTT/G	36,811,354	—	F: TCTCATGACCACAAACGAGTCCCT	135	—
					R: GGTCTTGGTGACCTTGACCATATGG		

**Fig. 8:**
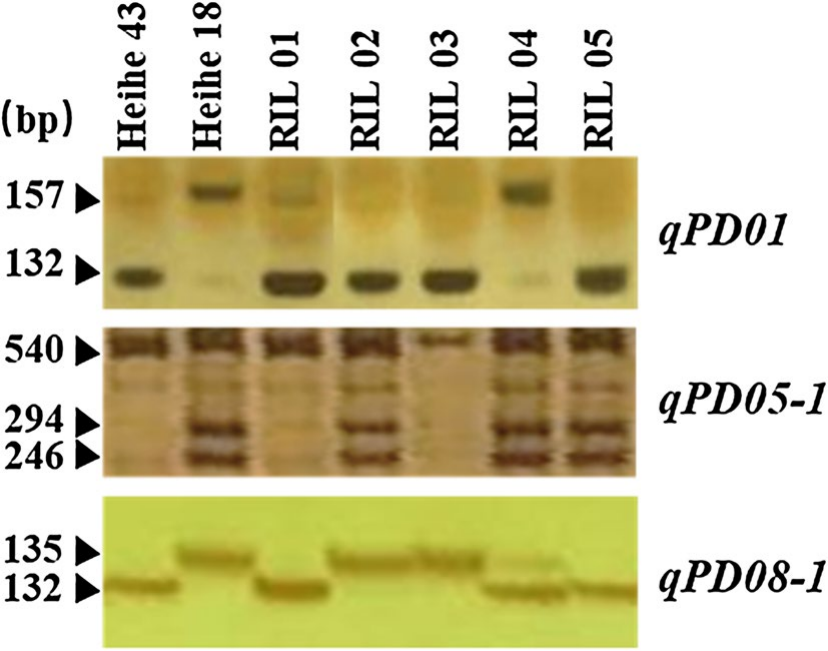
Validation of the molecular markers to QTLs *(qPD01*, *qPD05-1* and *qPD08-1*). Electrophoresis results of the polymerase chain reaction (PCR) of molecular markers for Heihe 43, Heihe 18 and five recombinant inbred lines (RILs)

After digestion with Mnl I, three digested products were produced theoretically by dCAPs marker in the *qPD01* interval. However, one fragment of 25 bp was too small to detect; therefore, only two kinds of digestion products were detected. The DNA fragment size without digestion was 157 bp, and the allelic variation was T. Material carrying the T allele showed the pod dehiscence. After digestion, there were two fragments, 132 bp and 25 bp. The allelic variation was C, and material carrying the C allele showed the resistance of pod dehiscence. Within the *qPD05-1* interval, the PCR products could be digested by HpaII and produced three digestion products theoretically. Only two kinds of digestion products could be detected due to the similar length of the two fragments after digestion. The DNA fragment size without digestion was 540 bp and the allelic variation was A that provided the resistance of pod dehiscence; the digested DNA fragment sizes were 294 bp and 246 bp and the allelic variation was G that provided the pod dehiscence. Within *qPD08-1*, we detected two bands: One DNA fragment size was 422 bp and the allelic variation was GTTT; the other DNA fragment size was 419 bp and the allelic variation was G that existed in the resistance of pod dehiscence. These markers were validated by using 260 RILs derived from two parents. The genotypes and phenotypes of five RILs are listed in Fig. 8 and Table 9. We found eight allele combinations in 260 RILs, and three combinations had more than ten individuals (Table 10). The identification efficiency to resistance of pod dehiscence for combination 1 with all three resistant alleles was the highest (97.4%), and both combination 2 and combination 4 with two resistant alleles were 84.2% and 82.4% respectively. The results indicated that the developed CAPS/dCAPS and Indel markers could be used to identify genotypes and predict the phenotypes of soybean varieties.

**Table 9:** The PD of the RILs used to identify the molecular markers

Year	PD (%)						
	Heihe 43	Heihe 18	RIL01	RIL02	RIL03	RIL04	RIL05
2015	3	37	0	68.8	56.3	34.4	9.5
2016	11.4	47	0	83.3	83.3	36.7	10
2017	3.3	75	0	83.3	83.3	36.7	10

**Table 10:** Identification efficiency of three molecular markers different allelic combination on resistant materials on 260 RILs

Combination	Allelic combination			No. of resistant materials	No. of susceptible materials	Total	PD (%)	Identification efficiency (%)
	*qPD01*	*qPD05-1*	*qPD08-1*					
1	C	A	G	189	5	194	7.0	97.4
2	C	G	G	16	3	19	20.2	84.2
3	T	G	G	5	1	6	15.0	83.3
4	C	A	GTTT	14	3	17	18.7	82.4
5	C	G	GTTT	6	2	8	27.8	75
6	T	A	GTTT	5	2	7	30.8	71.4
7	T	G	GTTT	2	5	7	41.4	71.4^a^
8	T	A	G	2	0	2	7.8	100

^a^71.4% indicates the identification efficiency to susceptible pod dehiscence

## Discussion

QTL mapping is an effective method for analyzing quantitative traits in soybean. The quality of a genetic map has a significant impact on the resolution of QTL mapping. For a specific population, increasing marker density improves the accuracy and efficiency of a genetic map (Gutierrez-Gonzalez et al. 2011; Yu et al. 2011; Zou et al. 2012). Nevertheless, both wild and cultivated soybeans exhibit high linkage disequilibrium (LD) (cultivated soybean: ~ 150 kb; wild soybean: ~ 75 kb), and the average genetic distance is greater than in other crops (Lam et al. 2010). Therefore, a high-density genetic map is needed to improve the efficiency and accuracy of QTL mapping in soybean and can be useful for molecular marker-assisted selection (Cao et al. 2017; Staff 2014).

SLAF-seq is an efficient method for identifying QTLs and genotyping based on NGS. This technique is advantageous for large numbers of individuals due to its convenient library preparation procedure (Elshire et al. 2011). Compared with SLAF-seq, genome resequencing provides a more accurate view of the genome and captures large variations as well as small ones that may be missed by SLAF-seq. However, SLAF-seq is relatively low cost and can be used for mapping in large-scale populations (Li et al. 2014; Sun et al. 2013). SLAF-seq provides the best balance between sequencing cost and sequencing depth. SLAF-seq also obtains genome-wide variation sites at a lower cost and provides for researchers to select functional polymorphic markers, construct high-density genetic maps and further identify functional genes of important agronomic traits (Wei et al. 2017). This method has been extensively used to construct high-density genetic maps and identify major QTLs related to complex traits in several plants (Xia et al. 2015; Xu et al. 2015; Han et al. 2015a, b; Qin et al. 2015; Su et al. 2016). Pod dehiscence is one of the most important agronomic traits in crops and is directly related to yield. Therefore, in many crops, including soybean, it is vital to identify the QTLs related to pod dehiscence and the markers tightly linked to pod dehiscence. In soybean, several studies have found QTLs for pod dehiscence located on chromosome 16 and have performed fine mapping using different plant materials (Bailey et al. 1997; Dong et al. 2014; Funatsuki et al. 2014; Gao and Zhu 2013; Hideyuki et al. 2012; Kang et al. 2009), but only two genes for these pod dehiscence QTLs have been cloned. Therefore, we detected novel QTLs associated with pod dehiscence in soybean using SLAF-seq and predicted candidate genes within QTLs based on the high-density genetic maps, which could be applied for identifying new QTLs for pod dehiscence and other valuable agronomic traits. The QTLs for pod dehiscence also provided promising candidate genes for further characterization.

### Constructing a high-density genetic map

Because of multiple generations of self-fertilization during the process of development of RIL populations, the observed recombination of RIL populations is greater than that of the corresponding F_2_ population. The increased recombination for a reference population that consists of RILs, when compared to an F_2_ reference population improves the accuracy and efficiency of the map. Moreover, in map-based cloning, secondary mapping populations are usually mapped after primary populations; however, doing so is laborious and time-consuming. In this study, we constructed a high-density genetic map and sequenced the whole genome in soybean RIL_7_ using SLAF-seq. High-quality markers were evenly distributed on 20 chromosomes. Recently, Cao et al. (2017) and Li et al. (2014) used SLAF-seq to construct high-density soybean genetic maps using 236 RILs and 200 RILs, respectively. These two genetic maps contained 3255 and 3541 SLAF markers, respectively, and the average genetic distances between two markers were 0.66 cM and 0.72 cM, respectively. Researchers have shown that increasing population number and marker density can significantly improve the efficiency of QTL mapping (Li et al. 2014). In our study, we used 260 RILs to construct a high-density genetic map containing 4593 SLAF markers. Compared with the genetic maps of Cao et al. (2017) and Li et al. (2014), the genetic distance between two adjacent SLAF markers was reduced to 0.53 cM in our map. Each chromosome contained 230 markers on average, and the shortest genetic distance between adjacent markers was 0.11 cM. Our high-density soybean genetic map may be due to the large size of the population, the increased number of SLAF markers and narrowed gaps among markers, which showed that our genetic map is an effective reference for soybean and can be used for further genetic mapping and prediction of pod dehiscence-related genes.

### QTLs for pod dehiscence

The trait of pod dehiscence has considerable genetic diversity in soybean cultivars (Caviness 1965; Tsuchiya 1986; Helms 1994; Romkaew and Umezaki 2006; Yamada et al. 2009). Tsuchiya (1986) predicted that three genes control pod dehiscence. Several studies have identified two major genes controlling pod dehiscence, named *qPDH1* and *SHAT1-5*, which are located on chromosome 16 (Bailey et al. 1997; Dong et al. 2014; Funatsuki et al. 2014; Gao and Zhu 2013; Hideyuki et al. 2012; Kang et al. 2009). Other minor QTLs related to pod dehiscence are located on chromosomes 2, 5, 10, 14, 15 and 19 (Bailey et al. 1997; Kang et al. 2009). In China, Luo et al. (2012) used 112 RILs and the composite interval mapping method and reported a QTL controlling pod dehiscence located in chromosome 6 named *qPDH6-1*.

Kang et al. (2009) identified seven minor QTLs for pod dehiscence using two RIL populations. Compared to this genetic map, our genetic map was constructed using a specific RIL population, which allowed us to construct a more efficient QTL map for a given phenotypic trait. We identified six novel QTLs for pod dehiscence on chromosomes 1, 5, 8 and 14 that were not detected in previous studies (Table 2). Of these six QTLs, *qPD05-1* and *qPD05-2* were located on chromosome 5, the same chromosome as a previously reported minor QTL (Satt 385) (Kang et al. 2009). In the USDA (United States Department of Agriculture) map, *qPD05-1* is located between Sat_374 and BARCSOYSSR_05_1304, and *qPD05-2* is located between BARCSOYSSR_05_0375 and BARCSOYSSR_05_0374. Interestingly, *qPD05-1* and *qPD05-2* were not at the same position as QTL Satt 385. Meanwhile, *qPD05-1* could be found across 2 years (2015, 2016), of which the LOD and the phenotypic variation reached maximum values of 11.19% and 15.14%, respectively. Therefore, this QTL could be identified as a major, stable QTL for pod dehiscence. The other QTL, *qPD14*, was located on chromosome 14, the same chromosome as a previously reported minor QTL (Satt 577) (Kang et al. 2009). Similar in the previous case, *qPD14* was located between Satt 063 and Sat_424. These two QTLs were not at the same position at chromosome 14.

As given in Table 2, the QTLs *qPD01* and *qPD08-1* could also be detected in different environments with phenotypic variation of 24.44% and 19.61%, respectively. Therefore, in our population, *qPD01*, *qPD05-1* and *qPD08-1* might be the major QTLs for pod dehiscence. *qPD05-2*, *qPD08-2* and *qPD14* were detected in a single environment; the phenotypic variation explained by *qPD05-2* was 17.57%, *qPD08-2* was 8.37%, and *qPD14* was 7.22%. QTLs detected in the progeny could be considered stable in different environments. Therefore, these QTLs and closely linked SLAF markers can be used for molecular marker-assisted selection in soybean.

It is interesting to note that the previously reported major QTLs on chromosome 16 were not identified in our study in all environments; this result might be explained by the different genetic background between the parents of the mapping populations; the same phenomenon was found in the other trait (Li et al. 2014). The QTLs that have been identified for pod dehiscence QTL such as *Pdh1* (Funatsuki et al. 2014) may be present in elite populations developed from crossing parents that are used for commercial soybean varieties including the parent we used Heihe 43, but we did not map this locus in our population. However, the QTLs that we have identified by crossing two parents that also used for developing commercial soybean varieties may have different mechanism of pod dehiscence resistance. Heihe 43 is the largest planted variety in Heilongjiang province of the People’s Republic of China at present; therefore, the breeding value of the QTLs we identified for pod dehiscence resistance can be contributed to genetic research of pod dehiscence in soybean.

### Gene annotation and putative candidate genes for pod dehiscence

We predicted candidate genes within a narrow region between two adjacent markers based on high-density genetic maps and the high-quality genome sequences of Williams 82. Gene prediction depends on high accuracy and collinearity between the genetic map and the reference genome (Dan et al. 2016). The high collinearity of the markers and genetic map in our map allowed us to identify gene candidates related to pod dehiscence within QTLs (Fig. 4). Gene annotation within six QTLs was performed using Nr, Swiss-Prot and KOG/COG databases. These QTLs contained 34 protein-encoding genes, of which 32 have GO annotations (Table 6). In order to obtain more information of candidate genes in pod dehiscence, the expression profiles of 31 genes (Fig. 7) between Heihe 43 and Heihe 18 were analyzed by qRT-PCR. Among 31 genes, nine genes had relatively high expression levels, and seven of nine genes had significant differences between two parents, including *Glyma.01g045800*, *Glyma.01g046000*, *Glyma.05g005600*, *Glyma.05g225900*, *Glyma.05g227400*, *Glyma.08g271900* and *Glyma.08g274500*. Previous studies have suggested that some enzymes control cell wall degradation (Christiansen et al. 2002) and synthesis (Romkaew et al. 2008). In our study, four genes involved some enzymes including fatty acid/sphingolipid desaturase (*Glyma.01G046000*), DNA methyltransferase (*Glyma.05G005600*), NADP-dependent malic enzyme (*Glyma.05G227400*) and oxidoreductase (*Glyma.08G274500*), but the function of these genes needs to be verified. Our results provided information for future study.

In this study, we constructed a high-density genetic map containing 4593 SLAF markers using 260 RILs. Our finding showed that this map was accurate and efficient for QTL mapping. We identified three stable QTLs associated with pod dehiscence resistance in three environments. These novel and stable QTLs not only contribute to study of the genetic mechanism of pod dehiscence but also facilitate molecular marker-assisted selection to increase production in soybean.

#### Author contribution statement

JH conducted the data analysis, QTL mapping, genomic comparative analysis and wrote the manuscript. DH and HY conducted the RIL populations and phenotyping. YG, ZW and YT provided advice on experimental implementation. LQ conceived and supervised the project, reviewed and revised the manuscript.
